# Nonlinear preprocessing method for detecting peaks from gas chromatograms

**DOI:** 10.1186/1471-2105-10-378

**Published:** 2009-11-18

**Authors:** Byonghyo Shim, Hyeyoung Min, Sungroh Yoon

**Affiliations:** 1 School of Information and Communication, Korea University, Seoul 136-713, Korea; 2 College of Pharmacy, Chung-Ang University, Seoul 156-756, Korea; 3 School of Electrical Engineering, Korea University, Seoul 136-713, Korea

## Abstract

**Background:**

The problem of locating valid peaks from data corrupted by noise frequently arises while analyzing experimental data. In various biological and chemical data analysis tasks, peak detection thus constitutes a critical preprocessing step that greatly affects downstream analysis and eventual quality of experiments. Many existing techniques require the users to adjust parameters by trial and error, which is error-prone, time-consuming and often leads to incorrect analysis results. Worse, conventional approaches tend to report an excessive number of false alarms by finding fictitious peaks generated by mere noise.

**Results:**

We have designed a novel peak detection method that can significantly reduce parameter sensitivity, yet providing excellent peak detection performance and negligible false alarm rates from gas chromatographic data. The key feature of our new algorithm is the successive use of peak enhancement algorithms that are deliberately designed for a gradual improvement of peak detection quality. We tested our approach with real gas chromatograms as well as intentionally contaminated spectra that contain Gaussian or speckle-type noise.

**Conclusion:**

Our results demonstrate that the proposed method can achieve near perfect peak detection performance while maintaining very small false alarm probabilities in case of gas chromatograms. Given the fact that biological signals appear in the form of peaks in various experimental data and that the propose method can easily be extended to such data, our approach will be a useful and robust tool that can help researchers highlight valid signals in their noisy measurements.

## Background

**Figure 1:**
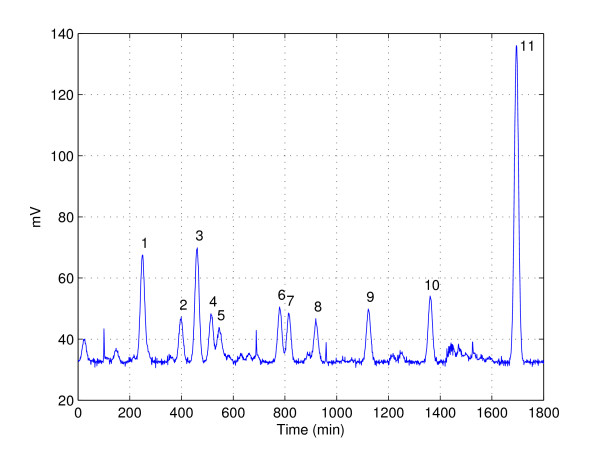
**Plot of GC data **[1]. Number is labeled on top of each true peak. According to [1], samples were analyzed on a Varian 3400 GC (Varian Instrument, Palo Alto, CA) equipped with a 100 m × 0.32 mm SP2380 (Supelco Inc., Bellefonte, PA) capillary column and flame-ionization detector (FID); helium was used as carrier gas.

When experimental observations are made, noise is inevitably introduced by instruments and surrounding environments. Needs for detecting peaks in the presence of noise thus occur frequently when analyzing experimental data. Valid signals sometimes appear in the form of peaks, and for accurate analysis of the observations made, researchers want to separate true peaks from fictitious peaks generated by noise. For instance, peak detection is considered critical in analytical chemistry in which the objective is to separate, identify and quantify sample compounds by using techniques such as gas chromatography (GC) and mass spectrometry (MS). Figure 1 shows an actual GC data set [1] that contains a great deal of false peaks resulting from instrumental noise. Another example can be found in a recent study called multiplexed hydroxyl radical (• OH) cleavage analysis (MOHCA), which is to predict the helical arrangements of large RNA molecules in a high-throughput manner [2]. Since incorrectly introduced false peaks have adverse effects on the subsequent procedures thereby resulting in a mislead conclusion, it is critical to choose the right peak corresponding to the true chemical.

In order to filter out false peaks and selectively detect valid ones, several preprocessing techniques such as thresholding or lowpass filtering (LPF) have been employed [3]. However, in many situations, these approaches either filter out valid peak information or fail to reject false peaks. Additionally, choosing appropriate parameters (e.g., cutoff frequency, rejection threshold) is mostly based on trial-and-error, so it is not uncommon to manually adjust these parameters. Indeed, since the peak detection is a blind problem and no prior knowledge on the information is given, tuning parameters has been a nontrivial task causing high false alarm and misdetection rates.

In this paper, we propose a novel peak detection method that is much less sensitive to parameter choices than conventional techniques, yet produces a very robust and accurate detection performance on noisy experimental data. The key feature in our method is a nonlinear preprocessing to suppress the noise and to strengthen the peak signal. Two major ingredients achieving this objective are geometric mean filtering (GMF) and wavelet domain denoising. A function of multiplying the observation and the reference signal for achieving correlation gain has been widely used in communication and signal processing [4]. Since no desired reference is available for the peak detection problem, the shifted version of experimental signal itself can serve as an approximate reference to obtain a coherent amplification of the peak signal.

Furthermore, since the output of GMF suppresses randomly fluctuating noise better than the conventional arithmetic-mean based filtering, the proposed GMF effectively differentiates the peak information and the noise. Once the GMF is finished, further cleaning of noise i.e., a denoising operation, is performed in the wavelet domain. Due to the increased frequency resolution of wavelet transform in low frequencies compared to the Fourier transform [5], peak information frequencies can easily be localized by their energy, and thus the noise and unwanted signal can nicely be separated from the peak. It is worth pointing out that there is a close relationship between the proposed approach and the denoising studies in image processing [6,7] in the sense that both filter out noise in the wavelet domain. While the image denoising needs to consider the low-energy wavelet coefficients for preserving the image shape, such is unnecessary for the peak detection problem since the unique goal is the identification of peak points. In fact, since the peak information is rarely located in wavelet coefficients with small energy, cleaning of those coefficients will rather help, in particular, to suppress sharp and narrow-shaped false peaks so-called *speckles*. When the GMF and wavelet domain denoising are finished, we clearly observe the suppression of the noise magnitude. Due to this clear distinction between the signal and noise, employing a nonlinear operation (amplification followed by slicing) can remove substantial amount of noise and hence facilitate the peak collection operation.

We test the proposed method with gas chromatography data and show that the proposed approach exhibits excellent peak detection performance with small false alarm probabilities. Further, we demonstrate the robustness of the proposed method using the extended scenario in which artificial noise, viz. speckles and Gaussian noise, is added into the data.

## Proposed method

**Figure 2:**
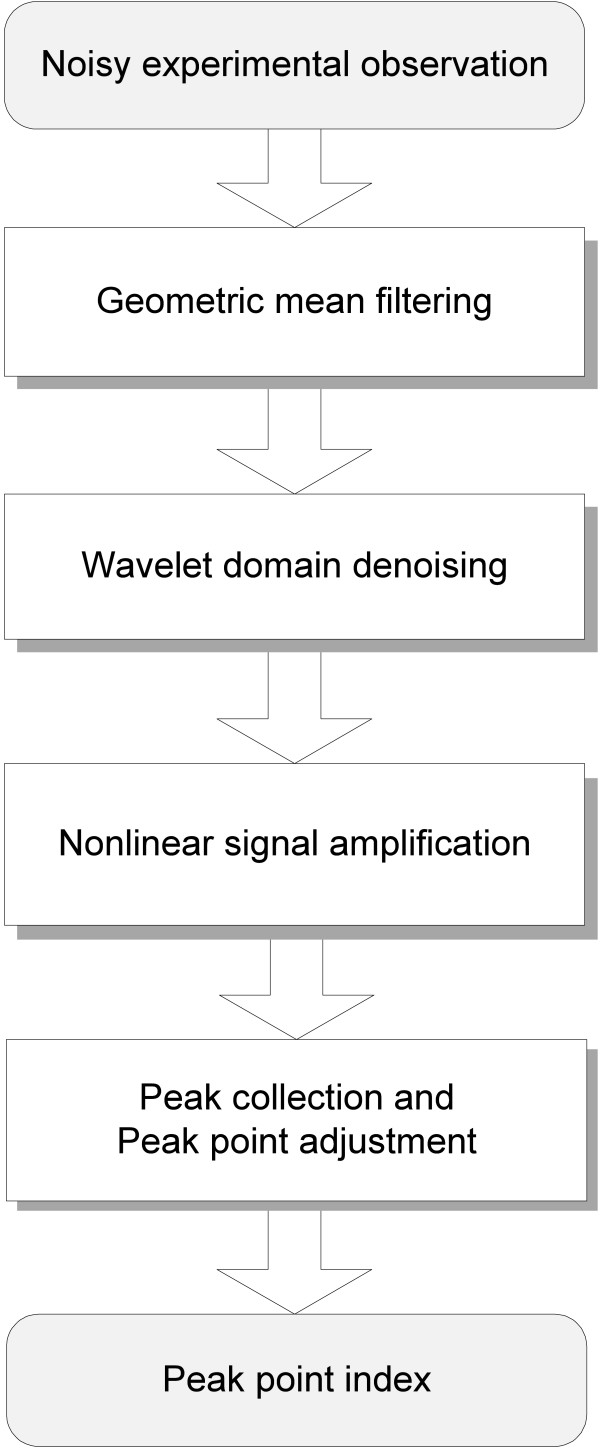
**Overview of the proposed method**. The proposed method consists of four major steps: (1) geometric mean filtering, (2) wavelet domain denoising, (3) Nonlinear signal amplification, and (4) peak collection and adjustment.

In this section, we briefly discuss the peak signal model used and then present the proposed preprocessing operations. The proposed preprocessing consists of three major steps: 1) GMF, 2) wavelet domain denoising, and 3) nonlinear amplification. Although these operations look independent, they are tightly correlated together for achieving the common goal. When the preprocessing operations are finished, zero-crossing-based peak collection [8] is finally performed in order to reap the detected peaks. The overall steps of the proposed method are illustrated in Figure 2, and more details of each step will be described in the following subsections.

### Peak signal model

The discrete model for an experimental observation we use is(1)

where *g *[*n*] and *v *[*n*] are the peak and noise signal, respectively. In order to design a systematic peak detection method, we should rely upon minimum guidelines on the characteristics of peak signal to detect.

For this purpose, we employ the following assumptions on the peak signal *g *[*n*] and peak point *n*_*p*_.

**A.1) ***g *[*n*] is gradually changing in a local interval *I*_*p *_= {*n*_*p *_- *δ*, ⋯, *n*_*p *_+ *δ*} around peak point *n*_*p*_. That is, |*g*[*n*_1_] - *g*[*n*_0_]| <*ϵ*_0 _for adjacent values of *n*_1_, *n*_0 _∈ *I*_*p*_, where *ϵ*_0 _is a pre-defined small constant.

**A.2) **The magnitude of peak signal is highest in *I*_*p*_. In other words, *n*_*p*_, a valid peak point, should satisfy  where .

**A.3) ***g*[*n*] is monotonically increasing in the local interval [*n*_*p *_- *δ n*_*p*_) and monotonically decreasing in (*n*_*p *_*n*_*p *_+ *δ*].

Notice that, since we cannot discriminate the signal *g *[*n*] and noise *v *[*n*], the assumptions we described in **A.1) **and **A.3) **are rather conceptual. Further, we have no clue on *ϵ*_0 _and *δ*, which are observation dependent parameters. In spite of this, these assumptions play an important role in our preprocessing since they provide useful guidance on the algorithm design.

### Geometric mean filtering (GMF)

The first step in the preprocessing stage is the geometric mean filtering (GMF). The output of (2*k *+ 1)-tap GMF [*n*] for the input sequence *x *[*n*] is defined as(2)

For filtering out the noise from the data, the correlation between *x *[*n*] and shifted version *x *[*n *- *i*] is exploited. As an example, consider the 3-tap (*k *= 1) GMF filter. For notational convenience, we denote the value of *g *[*n*] at *n *= *n*_0 _by *g*_0 _and *v *[*n*] at *n *= *n*_0 _- 1, *n*_0_, and *n*_0 _+ 1 by *v*_-1_, *v*_0_, and *v*_1_, respectively.

Noting that *g *[*n*] is gradually changing by **A.1)**, the observations at *n*_0_, *n*_0 _- 1, and *n*_0 _+ 1 are(3)(4)(5)

are where *ϵ*_*i *_<*ϵ *_0 _for *i *= 1, 2. The GMF of *r *[*n*_0 _- 1], *r *[*n*_0_], and *r *[*n*_0 _+ 1] is(6)

Due to the random fluctuation on *v *[*n*], the coefficient associated with , which is essentially the sum of zero mean random variables, gets close to zero. Denoting this term by *δ*, (6) can be written as(7)

**Figure 3:**
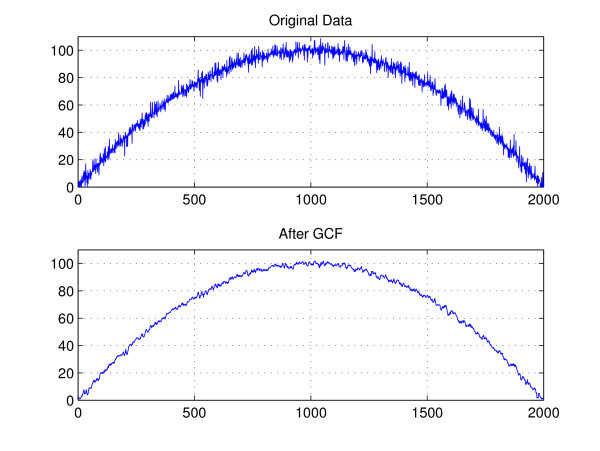
**Plots for *r *[*n*] and ****[*n*]**. Original data (*r *[*n*]) and after GCF ([*n*]).

As the filter tap increases, *δ *decreases and () comes close to unity, and thus [*n*] well approximates *g*_0_. Figure 3 illustrates the 5-tap GMF filter output for gradually changing function .

**Figure 4:**
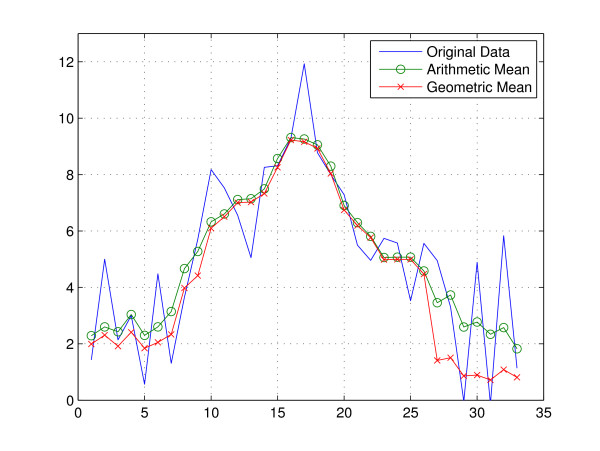
**Arithmetic mean ****[*n*] vs. Geometric mean ****[*n*]**. Although similar results might be obtained by the arithmetic mean filtering, for the region where the peak assumption is violated (e.g., the data is away from the peak), they becomes distinct.

Although similar results might be obtained by the arithmetic mean filtering, for the region where the peak assumption is violated (e.g., the data is away from the peak), they becomes distinct. Specifically, if *g *[*n*] ~*g*_0 _is dominant over *v *[*n*] in *I*_*p *_by the assumptions **A.1) **and **A.2)**, then *y *[*n*] ~*g*_0 _so that the arithmetic mean is similar to the geometric mean by the arithmetic-geometric mean inequality. However, for the samples in , *g *[*n*] is not dominant any more, and the geometric mean value becomes noticeably smaller than the arithmetic mean value in . Figure 4 illustrates this behavior for a randomly generated sequence. Owing to the function of lowpass filtering as well as the suppression of randomly fluctuating noise, the GMF output becomes more amenable to the subsequent denoising operation.

### Wavelet domain denoising

For further suppression of GMF filtered output, the wavelet transform is employed. The wavelet transform lends itself to separation between the signal and noise thanks to the inherent use of multi-resolution techniques by which different frequencies are analyzed with different resolutions [5]. In fact, it is far more efficient for extracting peak signals than the Fourier transform that only provides a constant resolution.

After passing through the GMF, [*n*] is readily modeled as(8)

where, contrary to **A.1)**, the filtered noise signal [*n*] is now limited globally, i.e., |[*n*]| <*ϵ*_0_. Thus, the noise power is clearly insignificant compared to the signal power and the signal and noise spectrum become more distinct in the transformed domain in their magnitude. In order to eliminate [*n*], we take the wavelet transform of [*n*] and then do the thresholding of the spectrum in the wavelet domain. Due to the enhanced frequency resolution in low frequencies, wavelet coefficients of the peak signal, mostly located in low frequencies, are well localized, and the wavelet coefficient of [*n*] becomes(9)

where (*k*), *G*(*k*), and (*k*) are the wavelet coefficients of [*n*], *g *[*n*], and [*n*], respectively. By applying the GMF function, the peak signal energy is compacted into a few wavelet coefficients, and the noise contributes mainly to the rest of insignificant coefficients. Hence, to denoise , we use the simplified assumption that low-energy coefficients are mostly due to the noise, whereas high-energy coefficients are mainly from the peak signal. A proper denoising strategy in this model is 1) to remove the low-energy wavelet coefficients substantially, and 2) to retain or modify slightly the high-energy coefficients. Although these assumptions might not strictly be true, they are sufficient for our purpose since the effect of small loss in the peak signal energy is minimal. For denoising  from , we use the soft-threshold estimator [9] given by(10)

**Figure 5:**
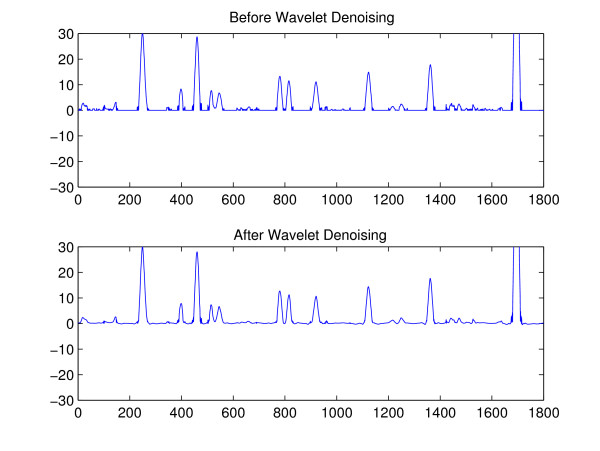
**GC data before and after the wavelet-domain denoising**. The GC data is already GMF filtered.

In Eq. (10), threshold *T*_*h *_is chosen as the *α*-percentile mean absolute of  given by(11)

where *I*_*α *_is the set of index *α *satisfying , and |*A*| is the cardinality of set *A*. As a trivial case, if *α *= 100, then *T*_*h *_returns to the mean absolute. The reason why we use the percentile mean is to control the threshold so that no valid peak signal is being erased. If some peak signal magnitude is very high, then the mean absolute value will also be large so that valid peaks with relatively small magnitude might be erased. We observe from the empirical test that 90 ~95 percentile generates satisfying results. Due to the removal of low-energy wavelet coefficients (|(*k*)| ≤ *T*_*h*_), the time-domain noise signal is suppressed substantially, as shown in Figure 5, and the signal after the inverse wavelet transform is safely modeled as(12)

where .

### Nonlinear signal amplification

As a final step of the preprocessing stage, nonlinear signal amplification of [*n*] is applied. Nonlinear signal amplification refers to the magnification of peak and the annihilation of noise signals followed by slicing. This operation is useful since it provides the last cleanup of the residual noise before the final peak collection step.

Since the peak collection is done via the zero-crossing operation after the derivative, of note is that the differential operator might amplify abrupt noise fluctuations, even though the magnitude of noise is very small. In fact, it is one of the primary reasons for high false alarm rates.

**Figure 6:**
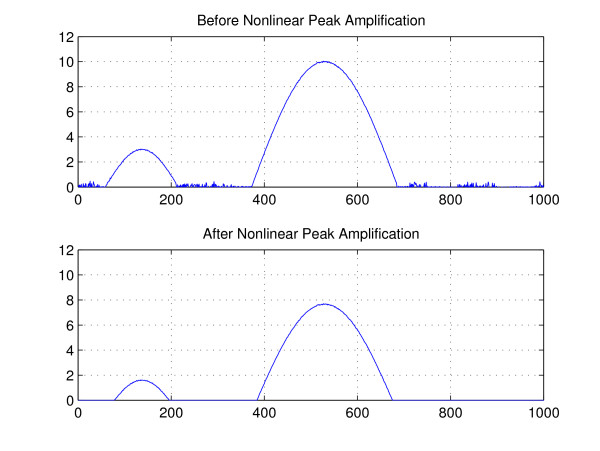
**Illustration of simulated data before and after the nonlinear signal amplification**. The noise with insignificant magnitude is removed by nonlinear signal amplification, thereby facilitating the subsequent peak collection step.

In order to prevent this behavior, it would be useful to clean up the small magnitude noise that can never be classified as a true peak. Towards this end, we use the following function(13)

where pos(*x*) = *x *for *x *≥ 0 and 0 otherwise. Clearly,  will amplify [*n*] greater than *E *[] and suppress [*n*] smaller than *E *[]. Because  for the pure noise (when *g *[*n*] = 0) and also , by the proper choice of *c*_0_, the argument inside the pos function becomes negative, ending up being zero after this pos function. For the choice of *c*_0_, it would be ideal to use . However, since  for most cases, 0.1 ≤ *c*_0 _≤ 1 would be enough as a simple choice. In Figure 6, we plot a result of Eg. (13) for simulated data with *c*_0 _= 1. As clearly shown in the figure, the noise with insignificant magnitude, which is mostly noise in real application, is removed thereby suppressing the false alarm rate significantly.

### Peak collection

In the absence of noise, the peak detection problem is equivalent to the problem of finding local maxima, and hence the points *t*_0 _satisfying  become the solutions for continuous-time signals *f*(*t*) [8]. In the case of discrete sequence *f *[*n*], the difference Δ_*f *[*n*] _= *f *[*n*] - *f *[*n *- 1] is being used instead of the derivative. Also, since no point satisfying Δ_*f *[*n*] _= 0 might exist, zero-crossing detection is indispensable. That is, if Δ_*f *[*n*] _> 0 and Δ_*f *[*n*+1] _< 0, *n *or *n *+ 1 is chosen as the peak point depending on their magnitude.

## Results and Discussion

### Test setup

In this section, we compare the performance of the proposed method with some conventional techniques including lowpass filtering (LPF) based preprocessing, wavelet domain thresholding (soft-thresholding [6] as well as hard-thresholding [9]), and pattern matching in continuous wavelet transform (CWT) domain (abbreviated to CWT method) [10]. For the proposed method and the wavelet domain thresholding, we employ the standard Cohen-Daubechies-Feauveau wavelet transform [11]. Defining the set containing the peak indices of original data *r *[*n*] as  and that of preprocessed data [*n*] as , the detection probability (*P*_*D*_) and the modified false alarm probability (*P*_*FA*_) are defined as(14)

and(15)

#### Real GC data

**Figure 7:**
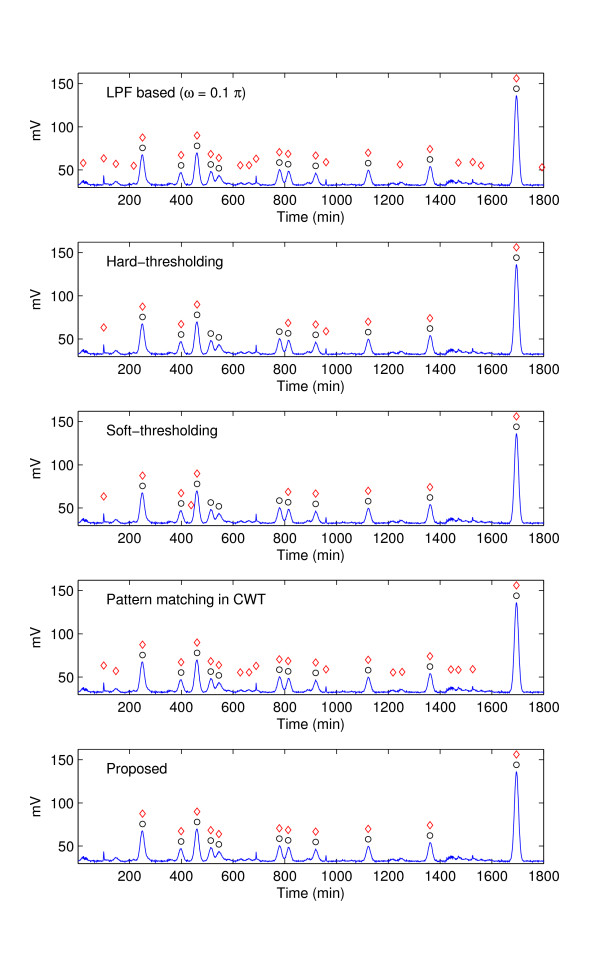
**Performance comparison (real data)**. The values in vertical axis represent *P*_*D *_and those in horizontal axis represent 10 × *P*_*FA*_. (See 'Real GC data' section for more details on the data used.)

In the test, 10 spectra obtained from an actual gas chromatography (GC) experiment were used for the performance comparison shown in Figure 7. Samples were analyzed on Acme 6100 GC with advanced pneumatic control (Young Lin Instrument Co, Korea). The analyzer is equipped with a HP Innowax capillary column (30 m × 0.53 mm, 1.0 *μ*m film thickness; Hewlett Packard, Palo Alto, USA) and flame-ionization detector (FID). Oven temperature program starting with a 15°C/min ramp from 150 to 180°C, followed by a ramp to 240°C at 5°C/min was employed. Helium was used as carrier gas. The flow rate was maintained at 3 ml/min for separations by using a mass flow controller, and the head pressure was set to 42.9 psi. The inlet temperature of the GC was 280°C. The injection volume was 0.2 *μ*l. The temperature of the FID was set to 280°C. System control and data evaluation were done using Autochro-3000 Data System. The minimum detection level (MDL) of the FID employed is less than 3.2 carbon pg/sec (dodecane) and the sensitivity is 19 mCoulomb/sec. It also has a linear dynamic range of 10^7^. The chromatograms used were measured at 0.00085 min intervals and each sample was analyzed for 14 min.

#### Simulated GC data

For checking the performance in noisier conditions, we additionally test the cases in which Gaussian and speckle-type random noise signals are intentionally added into the GC data used in [1]. As mentioned in the original article, samples were analyzed on a Varian 3400 GC (Varian Instrument, Palo Alto, CA) equipped with a 100 m × 0.32 mm SP2380 (Supelco Inc., Bellefonte, PA) capillary column and flame-ionization detector (FID). Helium was used as carrier gas.

#### Availability

The source code of the proposed method and the data used for validation are available at http://dna.korea.ac.kr/pub/gcpeak/.

### Experimental results

**Figure 8:**
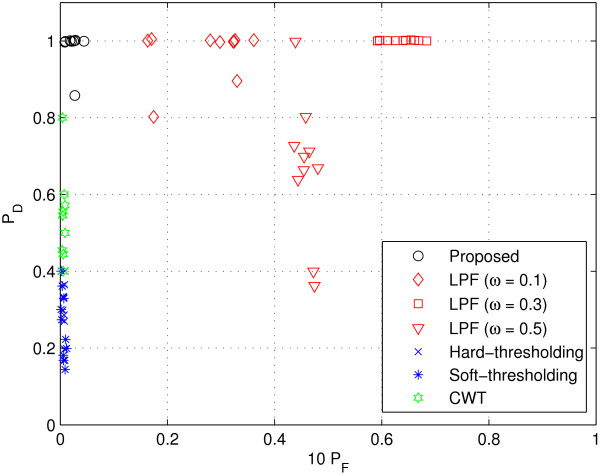
**GC data and the peak positions detected by each method**. Black circles indicate the original peak positions, and red diamonds correspond to the detected peak positions.

Figure 8 shows the test results from the GC data used in [1], where the *x*-axis in the curves indicates retention time (unit in minutes) and *y*-axis represents intensity (unit in millivolts). In the plot, 11 true peaks in the pre-determined positions are marked by black circles and the peak positions detected by each technique by red diamonds. Although the LPF-based processing shows good peak detection performance, we observe that this method detects too many invalid peaks resulting in high false peak probabilities. Since the thresholding in the wavelet domain without the proper distinction of peak signal and noise removes too much energy (including the energy of *g *[*n*]), the thresholding-based approaches tend to miss many valid peaks. Hence, even though the false alarm probability is moderate, this method is not desirable due to its poor detection performance. Interestingly, the proposed method is the best among the tested methods and displays the perfect performance (*P*_*D *_= 1 and *P*_*FA *_= 0).

**Figure 9:**
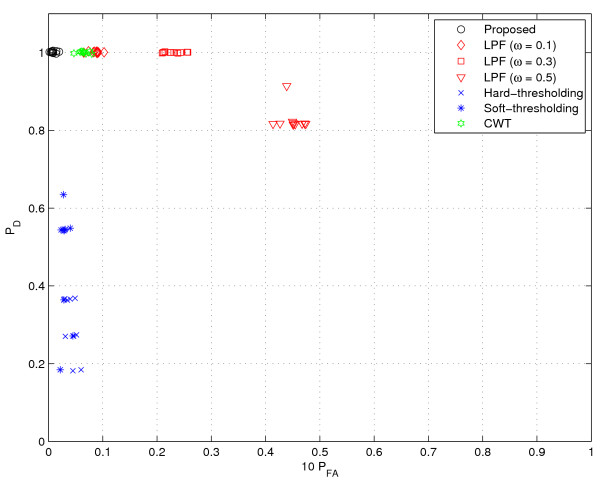
**Performance comparison (speckle noise I)**. || speckles are added.

**Figure 10:**
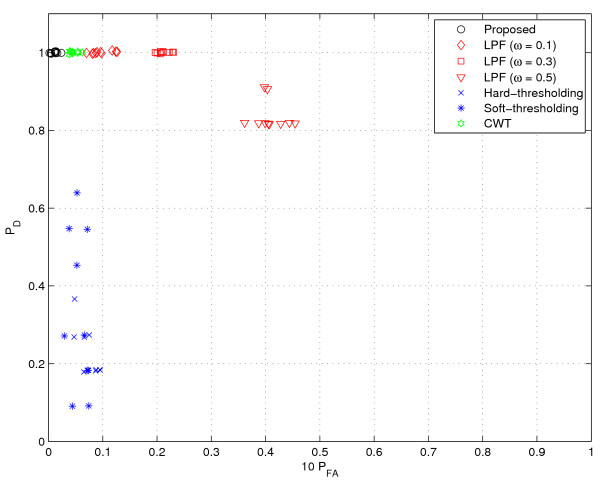
**Performance comparison (speckle noise II)**. 2|| speckles are added.

**Figure 11:**
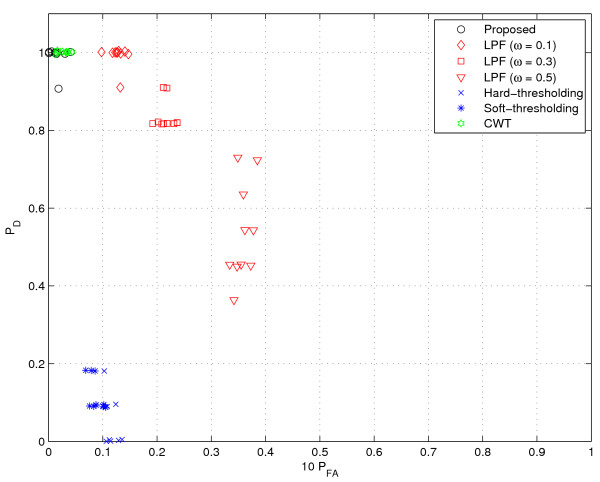
**Performance comparison (speckle noise III)**. 4|| speckles are added.

**Figure 12:**
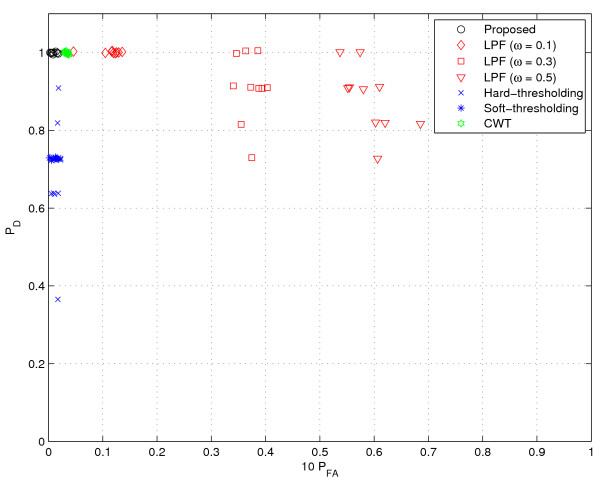
**Performance comparison (Gaussian noise I)**. 0.1% Gaussian noise.

**Figure 13:**
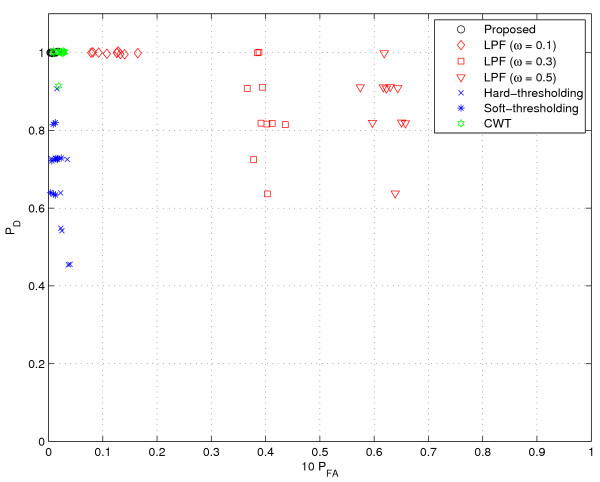
**Performance comparison (Gaussian noise II)**. 0.2% Gaussian noise.

**Figure 14:**
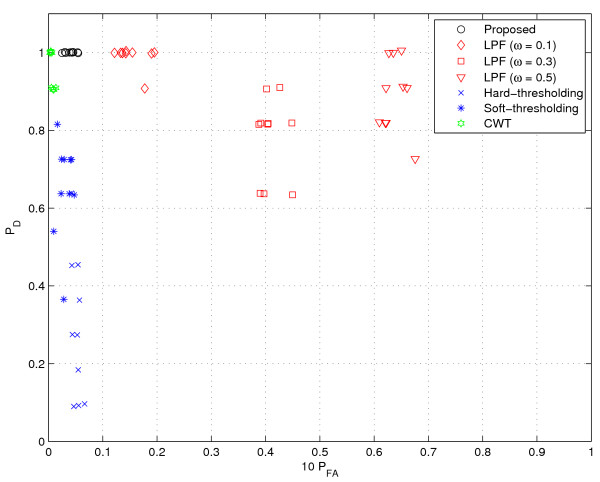
**Performance comparison (Gaussian noise III)**. 0.5% Gaussian noise.

To provide a comprehensive view on performance, we test the 10 spectra obtained from the GC experiments previously described (see 'Real GC data' above) and display a scatter plot where the values in vertical axis represent *P*_*D *_and those in horizontal axis represent 10 × P_*FA*_. Clearly, it would be the best if the data is located in the upper left corner (*P*_*D *_= 1 and *P*_*FA *_= 0). Notice that, since the number of data samples is much larger than that of valid peaks, *P*_*FA *_is very small even though the absolute number is considerable. To take this point into account, we display the scaled false alarm probability (10 × *P*_*FA*_) in the horizontal axis of Figures 8, 9, 10, 11, 12, 13 and 14.

As shown in Figure 8, CWT and thresholding techniques show slightly better false alarm probability than the proposed method but their detection performance is unsatisfactory. Whereas, the proposed method shows excellent *P*_*D *_while maintaining small *P*_*FA*_.

Next, we consider a scenario where speckle noise is added into GC data. In fact, this scenario models the instrumental noise from experimental devices or contamination due to impurities. To this end, we added speckle noise to the GC data from [1], which was described earlier in the 'Simulated GC data' section. In our experiments, we test three cases where the number of speckles are ||, 2||, and 4||, respectively. In addition, for some selected speckle points, we add the random noise chosen from the distribution *N*(*E *[*r*]; Var(*r*)). For each test case, we generate 10 random files. As shown in Figures 9, 10 and 11, we observe the general tendency that *P*_*D *_is getting worse as the noise level increases. The LPF method has a clear benefit in *P*_*D *_over the thresholding method. However, as the number of noise and the filter bandwidth increase, the performance degradation of the LPF technique becomes conspicuous. Whereas, the proposed method and CWT are insensitive to the speckle noise and provide excellent results. In particular, the proposed method is almost insensitive to the noise (*P*_*D *_= 1 for all 30 cases) and shows the best performance for all the tested cases.

Finally, we test a scenario where the GC data is corrupted by Gaussian noise. This scenario models the thermal noise of instrumental devices or temperature variations. We added Gaussian noise into the GC data from [1] described in 'Simulated GC data' above. In our experiments, we test three cases (0.1%, 0.2% and 0.5% of *E *[|*y*|^2^] as noise power) and generate 10 random files for each case. Due to the corruption of the whole data, as shown in Figures 12, 13 and 14, the performance degradation is in general much severer than that in the speckle type scenario. In particular, we observe degradation in *P*_*D *_of CWT as well as *P*_*FA *_of the LPF method. In contrast, the proposed method maintains the near perfect performance at 0.1% and 0.2% noise additions. Even at 0.5% noise addition, the proposed method outperforms all tested methods, providing perfect detection performance and small false alarm rates.

## Discussion

In this work, we addressed the problem of detecting peaks from noisy experimental data in a robust manner. The key ingredients of our approach to achieving this goal are geometric mean filtering (GMF), wavelet domain denoising, and nonlinear signal amplification. The GMF technique conducts the first round suppression of noise, and the wavelet domain denoising then performs the filtering of low-magnitude and high frequency noise. In the nonlinear signal amplification stage, noise clean-up is achieved by zeroing out the residual noise. From our experimental studies on the GC data, we observed that the proposed method shows near perfect peak detection and false alarm performance and that it is the best among the compared methods. Although the setup in this study is primarily for the GC data (including the extended tests with Gaussian and speckle-type contaminated GC data), we could observe that the proposed method can be extended to other types of experimental data as well. However, for the detection of speckle-type peaks appearing in, for instance, mass spectrometry experiments, the assumptions of the proposed method **(A.1) **and **A.3)**) need to be modified. In fact, regarding this extension, there are interesting directions worth pursuing. Our GMF relies only on data with integer delays. Hence, the result might not be desirable when the peak duration is very short, as in the case of speckles. In this case, it might be better to use non-integer delays by applying non-integer interpolated GMF. In addition, when the contamination level is severe, it would be a reasonable choice to use the cascade of the proposed method and supervised learning by which additional reliability might be inserted into the peak detection.

## Conclusion

We have devised a computational method for detecting signals appearing in the form of peaks from noisy experimental observations. Compared with previous techniques, the proposed method is unique in the sense that (1) it requires much less efforts to tune algorithm parameters and (2) its false detection rate is significantly lower, yet maintaining near perfect peak detection performance. We tested the proposed technique extensively with actual data obtained from gas chromatography experiments. In addition, in order to demonstrate the robustness of our approach, we deliberately incorporated two types of noise (speckle and Gaussian) into the original data and tested our technique with the data. In all the experimental studies we conducted, the proposed technique outperformed the alternatives we tried in terms of true and false positive rates and sensitivity to parameters. Given the fact that researchers are very much interested in isolating meaningful signals accurately from their experimental data in an automated and robust manner, we believe that the proposed method can lead to a significant contribution to the field.

## Competing interests

The authors declare that they have no competing interests.

## Authors' contributions

BS devised the algorithm, developed the code, performed the simulation and drafted the manuscript. HM prepared the experimental data, analyzed the result and drafted the manuscript. SY conceived and edited the manuscript. All the authors have read and approved the final manuscript.
